# Evaluation of the Antimicrobial Peptide, RP557, for the Broad-Spectrum Treatment of Wound Pathogens and Biofilm

**DOI:** 10.3389/fmicb.2019.01688

**Published:** 2019-07-24

**Authors:** Kathryn Wynne Woodburn, Jesse M. Jaynes, L. Edward Clemens

**Affiliations:** ^1^Riptide Bioscience, Vallejo, CA, United States; ^2^Integrative Biosciences, Tuskegee University, Tuskegee, AL, United States

**Keywords:** antimicrobial peptides, antibacterial, biofilm, bacterial resistance, multidrug resistance, wound infection

## Abstract

The relentless growth of multidrug resistance and generation of recalcitrant biofilm are major obstacles in treating wounds, particularly in austere military environments where broad-spectrum pathogen coverage is needed. Designed antimicrobial peptides (dAMPs) are constructed analogs of naturally occurring AMPs that provide the first line of defense in many organisms. RP557 is a dAMP resulting from iterative rational chemical structural analoging with endogenous AMPs, human cathelicidin LL-37 and Tachyplesin 1 and the synthetic D2A21 used as structural benchmarks. RP557 possesses broad spectrum activity against Gram-positive and Gram-negative bacteria and fungi, including recalcitrant biofilm with substantial selective killing over bacterial cells compared to mammalian cells. RP557 did not induce resistance following chronic passages of *Pseudomonas aeruginosa* and *Staphylococcus aureus* at subinhibitory concentrations, whereas concurrently run conventional antibiotics, gentamycin, and clindamycin, did. Furthermore, RP557 was able to subsequently eliminate the generated gentamycin resistant *P. aeruginosa* and clindamycin resistant *S. aureus* strains without requiring an increase in minimum inhibitory concentration (MIC) concentrations. RP557 was evaluated further in a MRSA murine wound abrasion infection model with a topical application of 0.2% RP557, completely eliminating infection. If these preclinical results are translated into the clinical setting, RP557 may become crucial for the empirical broad-spectrum treatment of wound pathogens, so that infections can be reduced to a preventable complication of combat-related injuries.

## Introduction

The continuing emergence of multidrug antimicrobial resistance and recalcitrant biofilm substantially hinders wound treatment. The U.S. military experience in Iraq and Afghanistan included an epidemic of wound infections featuring multidrug-resistant organisms (MDROs; Murray, 2008). Ballistic wound infection has become the greatest threat to the life and recovery of the combat casualty who survives the immediate trauma of the insult (Murray, 2008). Modern combat wounds are troublesome compared to peacetime traumatic injuries, because the higher velocity projectiles inflicted by improvised explosive devices (IEDs) cause more severe injury and accompanying wounds are readily contaminated by pathogens (Blyth et al., 2015). Ballistic wounding commonly inflicted by IEDs accounts for 75% of all recent war injuries (Owens et al., 2007). The widespread fragmentation associated with IED detonation and the changing microbial milieu place the casualty at risk of developing infections and place considerable burden on the military health care system. Innovation in the development of therapeutic agents with broad-spectrum anti-fungal and anti-bacterial activity, including MDROs and resilient biofilms, is urgently needed so that infections can be reduced to a preventable complication of combat-related injuries.

Combat wounds, in the absence of topical antibiotics, are immediately colonized by Gram-positive skin flora, such as *Staphylococcus aureus* (D'Avignon et al., 2008). Gram-negative bacteria such as *Pseudomonas aeruginosa, Klebsiella pneumoniae*, and *Escherichia coli*, from the patients' respiratory and gastrointestinal tract, typically colonize the wound 48 to 72 h post injury. *S. aureus* and *P. aeruginosa* are the culprit pathogens that are most likely to result in an invasive infection shortly after burn injury (D'Avignon et al., 2008; Pastar et al., 2013). Multidrug resistance is common, thereby limiting antibiotic therapy options (Petersen et al., 2007). Furthermore, there has been an increase in multidrug-resistant *Acinetobacter baumannii* in combat wounds of servicemen in Afghanistan and Iraq, hence the term “Iraqibacter” (Albrecht et al., 2006).

The combat environment moreover, with the ever-present threat of danger and a chaotic medical care environment with emergency triaging and possible onslaught of military and civilian casualties, makes adherence to stringent infection control practices difficult. Many pathogens have the ability to evade the effects of antimicrobial treatments due to development of resistance in addition to biofilm formation. Bacteria and fungi encased in biofilms are highly resistant to antibiotic treatment and the host's immunity, which in combination with the increasing prevalence of antibiotic resistance among human pathogens, further confounds treatment of biofilm-related infections (Akers et al., 2014; Olsen, 2015). The resistance of bacteria residing in biofilms is due to the biofilm matrix acting as a fortified barrier and the presence of slow-growing bacteria with poor metabolic activity, known as persisters (Wood, 2017).

Designed antimicrobial peptides (dAMPs) are laboratory synthesized peptides that are rationally designed analogs of naturally occurring AMPs that provide the first line of defense against invading pathogens in all multicellular organisms (Kumar et al., 2018). dAMPs have direct antibiotic activities in addition to modulating immune responses (Haney et al., 2019). dAMPs possess an amphipathic α-helix or β-sheet structure and a net positive charge, critical physicochemical features integral to electrostatically interacting and selectively perturbing the barrier function of the predominantly anionic pathogen membrane. Moreover, this remarkable targeting and direct contact disruption of the bacterial membrane makes bacterial resistance less likely to develop (Clemens et al., 2017; Hamoen and Wenzel, 2017). AMPs are now being recognized for their ability to target drug-resistant biofilms (de la Fuente-Núñez et al., 2016). The naturally occurring AMP, LL-37, inhibits biofilm and yet demonstrates minimal direct antibacterial activity (Haney et al., 2019).

The increasing emergence of antibiotic resistance also highlights the need for innovative alternatives that provide rapid and complete microbicidal activity with minimal safety issues, while exhibiting limited susceptibility to mechanisms of microbial resistance. Herein we evaluate RP557, a 17-amino acid dAMP that is the product of three iterative designed cycles of peptide synthesis followed by antimicrobial activity, cytotoxicity and proteolytic susceptibility evaluation. RP557 is being developed for the empirical treatment of combat wounds to prevent, and also treat, infections and in the civilian setting, the treatment of acute bacterial skin and skin structure infections.

## Materials and Methods

### Peptides

**Figure 1:**
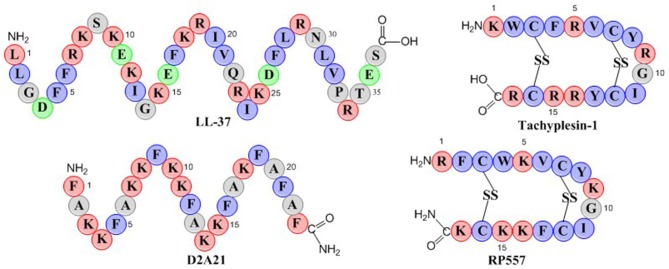
Schematic representation of the endogenous AMPs, LL-37 and Tachyplesin 1 and the dAMPs, D2A21 and RP557. Hydrophobic residues are in blue, while those residues with a net positive charge are in red and those with a negative charge are in green.

RP557 is a 17-amino acid peptide with two disulfide bridges as depicted in the schematic in Figure 1. RP557 was the product of three iterative designed cycles of peptide synthesis with the AMPs LL-37, D2A21, and Tachyplesin 1 peptides used as chemical benchmarks (Figure 1, detailed description surrounding the optimization of RP557 will be presented elsewhere). LL-37 is expressed endogenously in human phagocytic leucocytes and epithelial cells with elevated levels associated with wound healing, angiogenesis and regulation of inflammatory responses (Dürr et al., 2006). LL-37 exhibits minimal direct antibacterial activity however it effectively inhibits biofilm (Haney et al., 2019), therefore the evaluation of its requisite structural elements responsible for biofilm activity is important. Tachyplesin 1, a disulfide β-sheet AMP found in hemocytes of the horseshoe crab (*Tachypleus tridentatus*), a creature that has remained relatively unchanged for more than 450 million years, underscoring its successful ability to control infection (Nakamura et al., 1988). D2A21 is a synthetic dAMP derived from the cecropin family of α-helical AMPs that protect the Giant Silk Moth from bacterial infection and is being assessed for the topical treatment of chronic wounds (Chalekson et al., 2003).

The physiochemical properties of RP557 that have been optimized are shifts in the hydrophobic moment and modifying the centers of cationic charge of the disulfide disulfide β-sheet peptide configuration by exchanging arginine for lysine. The unique sequence of RP557 provides a relationship between net charge, amphipathicity and hydrophobicity that has resulted in decreased proteolytic degradation, broad spectrum antimicrobial and biofilm inhibition activity with reduced mammalian cytotoxicity and an increased safety index.

RP557 was synthesized via solid phase synthesis (AmbioPharm, North Augusta, SC, USA). Peptide purity was >96% as assayed by high performance liquid chromatography and mass spectroscopy. Reference peptides, LL-37 and D2A21 were obtained from Eurogentec (Fremont, CA) and AmbioPharm, respectively.

### Stability

To evaluate serum stability, 250 μL RP557 was incubated with pooled normal human serum at a concentration of 200 μg/mL for up to 72 h at 37°C. Samples, run in triplicate, were precipitated by addition of 0.3 g trichloroacetic acid, vortexed, neutralized with ammonium hydroxide (185 μL, 14.7 N) and purified and concentrated using C18 Spin Tips (ThermoFisher Scientific, Rockford, lL, USA); spin columns containing porous C18 reversed-phase resin. Freeze-thaw stability of RP557 was evaluated in rat plasma by analyzing six replicate 40 and 750 ng/mL samples after storage at −80°C for three freeze-thaw cycles with thaw cycles maintained at ambient temperature for a minimum of 1 h each. RP557 was evaluated using LC–MS/MS (AB Sciex 5500 Qtrap with a Shimadzu HPLC and data analyzed using Analyst software).

### Pathogens

Strains were obtained from American Type Culture Collection (ATCC, Manassas, VA) or the clinical isolate collection at Trideum Biosciences (Frederick, MD, USA). Bioluminescence strains of *P. aeruginosa* 19660, transfected with the Xen5 luciferase gene, and *S. aureus* ATCC® 49525™ (Wright), transfected with the Xen36 luciferase gene, were obtained from Perkin Elmer (Hopkinton, MA).

For Minimum Inhibitory Concentration (MIC) determination, bacteria and fungi were thawed from −80°C storage and sub-cultured on trypticase soy broth agar plates at 37°C prior to experimental evaluation. A single colony was aseptically picked from an agar overlay and released in 25–30 mL of pre-warmed trypticase soy broth in a culture flask. Each culture was incubated overnight in a 37°C incubator. Test agent stocks were serially diluted in water within 96-well plates, an overnight culture was diluted to approximately 10^6^ colony forming units (CFU)/mL using 2X cation adjusted Mueller Hinton broth and added to the test agent solutions (0, 2, 4,8, 16, 32, 64, and 128 μg/mL) for 24 h at 37°C. Pathogen growth was measured by absorbance at 600 nm. Four replicas were included for each test agent concentration. Each MIC was assigned to the lowest test agent concentration resulting in at least three out of four wells showing no growth based on an optical density (OD) reading of <0.1.

Biofilms (methicillin-resistant *S. epidermidis*, MRSE ATCC 51625 and multidrug resistant *S. epidermidis* MDR-SE ATCC 700578) were established following inoculation of 10^5^ CFU/mL in Minimum Essential Media for 6 h or 5 days representing preformed or mature biofilm respectively, in plastic 96-well plates (Costar, Catalog #3595). Unattached planktonic cells were removed, media replenished and the biofilm treated for 16 h with either RP557 or daptomycin. Following test agent incubation, the media (including the planktonic cells) was removed and then the remaining biofilm treated with 50 μL 0.1% Triton X100 followed by agitating the plate on a rocker for 30 min. The biofilm was then disrupted with a 96 well replicator, the resultant mixture vortexed, samples serially diluted, plated on agar plates, and incubated overnight and CFUs subsequently counted. Three technical replicates were performed for each condition tested.

### Time-Kill Assays

Non-invasive real-time evaluation of viability, mammalian, and bacterial cells, was performed using bioluminescent human keratinocytes (HaCaT, AddexBio, San Diego, CA) and murine L929 fibroblast cells (ATCC, Manassas, VA). The bioluminescent variants were constructed by transfection with a luciferase gene (RediFect Red-Fluc-Puromycin, Catalog #CL596002) (Hamblin et al., 2003). The resultant bioluminescent mammalian cells, and in separate experiments, bioluminescent *P. aeruginosa* or *S. aureus* cells (1 × 10^4^ cells, 100 μL) were plated in 96-well black-walled plates and RP557 was 2-fold serially diluted from 2,048 μg/mL in growth medium supplemented with 150 μg/mL D-luciferin to achieve a final volume of 200 μL. Each concentration was performed in triplicate. Bioluminescence imaging was performed at pre-specified times (0, 15, and 30 min and 1, 3, 5, 8, and 24 h) and compared to concurrently run vehicle-controls. The 96-well plate was positioned on the stage (12.5-cm field of view) within the *in vivo* Imaging System (IVIS) imaging system (Caliper Life Sciences, Inc., Hopkinton, MA, USA), with an open emission filter, binning of 4, and f-stop 1, and a 1-min exposure time. The resultant photons emitted from the live cells were quantified using Living Image® software (version 4.3, Caliper Life Sciences, Inc.). Monitoring bioluminescence allows assessment of the kinetics of cellular viability as there is a tight correlation between photon count imaging and viable counts (Kadurugamuwa et al., 2003).

### Antibiotic Resistance Profiling

Sub-inhibitory concentrations of RP557, gentamicin and clindamycin were incubated with *P. aeruginosa* ATCC 27853 and *S. aureus* ATCC 29213 for 24 h. The bacteria exhibiting growth in the highest concentration were then re-passaged in fresh dilutions containing sub-MIC levels of each test agent for 30 consecutive passages. The development of resistance was confirmed if the microorganism started growing at continuously higher concentrations. Following the 30-serial passaging resistance profiling, the subsequently generated gentamycin-resistant *P. aeruginosa* ATCC 27853 and clindamycin-resistant *S. aureus* ATCC 29213 strains were treated with 2 μg/mL RP557 to confirm RP557 susceptibility. Evaluations were done in duplicate.

### Human Hemolysis Assay

Human red blood cells were incubated with varying concentrations (0.25, 0.5, 1, 2, 4, 8, 16, 32, 64, and 128 μg/mL) of RP557, D2A21, and amphotericin b for 30 min at 37°C. Red blood cell lysis was indicated by extracellular soluble hemoglobin measured as absorbance at 540 nm. Distilled water was used as a control for 100% hemolysis with the background level of absorbance being water only (Stasiuk et al., 2004). The percent hemolysis was calculated as (test well_Abs_ – background_Abs_) / (100% lysis_Abs_ – background_Abs_). Experiments were run in duplicate.

### *In vivo* Murine MRSA Wound Infection Model

The murine MRSA needle scratch model, developed by Dai et al. (2010) utilizing the bioluminescent MRSA Xen31 (Xenogen Corp., Alameda, CA), derived from *S. aureus* ATCC 33591 was utilized. Bioluminescent MRSA (Xen 31) culture was prepared by growing overnight in brain heart infusion at 37°C with 100 rpm orbital shaking. Bacterial growth was assessed with a spectrophotometer. Optical density (OD) at 600 nm of 0.6 corresponded to a bacterial cell density of 2.7 × 10^7^ CFU/mL (Xen 31). Cells were washed and re-suspended in phosphate buffered saline (PBS) and used at a density of 10^8^ CFU/mL.

Seven-week old female BALB/c mice (Charles River Laboratories, Wilmington, MA) weighing 15–21 g, were intraperitoneally administered cyclophosphamide 4 days and 1 day prior to MRSA inoculation. The first dose was 150 mg/kg with the second being 100 mg/kg. Cyclophosphamide was administered so as to reduce the peripheral blood neutrophils to <100/μL blood, enabling the animal's vulnerability to infection and also reducing the recovery time (Vecchio et al., 2013).

Skin abrasion wounds were introduced on the shaved dorsal surface following anesthetization with 2.5% isoflurane. The scratches were applied in a 6 × 6 crossed design covering an area of 1 cm^2^. Care was taken not to draw blood and to confine damage to the stratum corneum and upper-layer of the epidermis. Five minutes following wounding, 40 μL of 10^8^ CFU/mL bioluminescent MRSA (Xen 31) in PBS, was inoculated over the wounded scratched area producing a total inoculum of 4 × 10^6^ CFU.

Four hours post infection, animals were divided into two groups, 6 animals per group, with one group serving as the infected control while the other, the treatment group. For the treatment group, 40 μL of 0.2% RP557 formulated in hydroxypropyl methylcellulose (2% in water viscosity 3,000–5,600 cP, VWR 75811-344 25G) was applied directly onto the infected wound, following which a transparent film dressing measuring 0.75 × 0.75 cm (Tegaderm™, 3M, St. Paul, MN) was placed over the wound area. To ensure the groups were treated equally, the film was also applied to the untreated group. To ensure animals did not interfere with each other's wounds, the mice were singly caged. Animals were weighed daily and clinical observations recorded.

Animals were imaged daily from Day 0 (application of bacteria) to Day 10. Animals were anesthetized with 2.5% isoflurane and placed dorsal side up in the IVIS chamber, imaging parameters included open filter, bin 4, 12.5 cm field of view, and f-stop 1.

### Statistical Analyses

Data is expressed as mean ± standard deviation or standard error. The significance of the results was determined using an unpaired two-tailed *t*-test for pairwise comparisons or ANOVA with the Tukey *post-hoc* pairwise *t*-test for comparisons of three or more groups using GraphPad Prism version 8 for Windows, GraphPad Software, San Diego, California USA, www.graphpad.com. *P* < 0.05 was considered significant.

## Results

### Robust Stability

**Figure 2:**
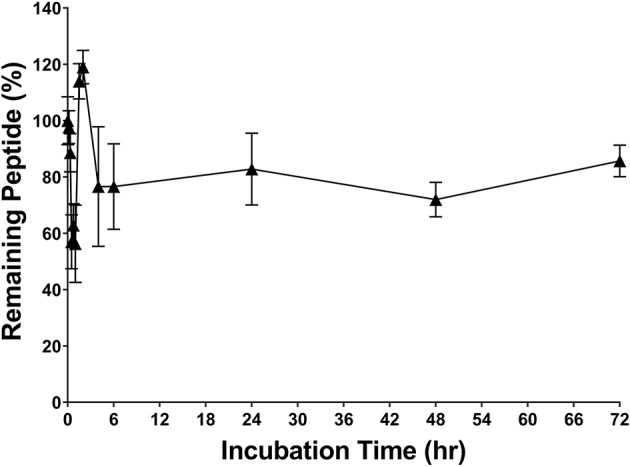
RP557 is stable in serum. RP557, at 200 μg/mL, was incubated with human serum for 72 h at 37°C and peptide monitored via LC-MS/MS. Data represent mean ±SE. Assays were performed in triplicate.

RP557 was the product of three iterative rationally designed cycles of peptide synthesis with the naturally occurring AMPs, human cathelicidin LL-37 and Tachyplesin 1 and the synthetic D2A21 used as chemical dAMP benchmarks (Figure 1). RP557 is conformationally locked by two disulfide linkages, that are integral to its enhanced activity and stability. To underscore RP557's enhanced stability, RP557 did not degrade following incubation for 72 h at 37°C underscoring its resistance to proteolysis; The serum stability profile of RP557 in human serum, with peptide monitored via LC-MS/MS is displayed in Figure 2. Furthermore, RP577 did not show signs of degradation following three freeze-thaw cycles.

### Broad Spectrum Antimicrobial Activity

RP557 possesses broad-spectrum antimicrobial activity against multiple clinical isolates of both Gram-negative bacteria and Gram-positive bacteria, including drug resistant phenotypes and fungi (Table 1). As expected, RP557 displayed superior antimicrobial activity against all clinical isolates compared to LL-37. In comparison, to Tachyplesin 1, RP557 had better activity against Gram-negative isolates, slightly better against Gram-positive isolates and consistently better microbiocial activity against fungi. With respect to clinically used comparators such as vancomycin, RP557 exhibited superior activity against Gram-negative isolates and *S. aureus* B-767. In addition, when compared to tobramycin, RP557 generally had better activity against Gram-negative isolates.

**Table 1:** Minimum Inhibitory Concentration (MIC, μM, [μg/mL]) evaluation of RP557, LL-37, tobramycin and vancomycin against planktonic gram-positive and gram-negative bacteria and fungi.

**Bacteria**	**RP557**	**Tachyplesin-1**	**LL-37**	**Tobramycin**	**Vancomycin**
**Gram-negative isolates**					
*A. baumannii* 6043	4.0 [8]	7.1 [16]	14.2 [64]	68.5 [32]	>88.3 [128]
*A. baumannii* 6838	4.0 [8]	14.1 [32]	>28.5 [128]	4.3 [2]	>88.3 [128]
*A. baumannii* ATCC 17978	8.0 [16]	3.5 [8]	28.5 [128]	4.3 [2]	>88.3 [128]
*E. cloacae* 6053	4.0 [8]	3.5 [8]	>28.5 [128]	68.5 [32]	>88.3 [128]
*E. cloacae* 6054	4.0 [8]	1.8 [4]	>28.5 [128]	137 [64]	>88.3 [128]
*K. pneumonia* 6066	4.0 [8]	28.2 [64]	>28.5 [128]	8.6 [4]	>88.3 [128]
*K. pneumonia* 6069	2.0 [4]	7.1 [16]	>28.5 [128]	4.3 [2]	>88.3 [128]
*K. pneumonia* ATCC 10031	4.0 [8]	3.5 [8]	>28.5 [128]	4.3 [2]	>88.3 [128]
*P. aeruginosa* 6186	2.0 [4]	3.5 [8]	>28.5 [128]	>274 [128]	>88.3 [128]
*P. aeruginosa* ATCC 19660	2.0 [4]	3.5 [8]	28.5 [128]	>274 [128]	>88.3 [128]
*P. aeruginosa* ATCC 27853	2.0 [4]	1.8 [4]	28.5 [128]	4.3 [2]	>88.3 [128]
**Gram-positive isolates**					
*S. aureus* B-767	2.0 [4]	7.1 [16]	>28.5 [128]	>274 [128]	>88.3 [128]
*S. aureus* 6061	4.0 [8]	3.5 [8]	>28.5 [128]	8.6 [4]	2.1 [3]
MRSA 6313	2.0 [4]	3.5 [8]	>28.5 [128]	>274 [128]	1.4 [2]
MRSA 6381	2.0 [4]	3.5 [8]	>28.5 [128]	274 [128]	1.4 [2]
MRSA ATCC 33592	2.0 [4]	3.5 [8]	>28.5 [128]	137 [64]	1.4 [2]
*S. epidermis* ATCC 51625	4.0 [8]	1.8 [4]	>28.5 [128]	4.3 [2]	2.1 [3]
**Fungi**					**Amphotericin b**
*C. albicans* Y-326	8.0 [16]	14.1 [32]			1 [1.08]
*C. albicans* Y-6359	2.0 [4]	14.1 [32]			1 [1.08]
*C. parapsilosis* Y-1761	8.0 [16]	28.2 [64]			1 [1.08]
*C. parapsilosis* Y-1763	1.0 [2]	7.1 [16]			1 [1.08]

*MIC is the lowest concentration of test article that shows no growth after a 24-h incubation. Data represent the mean of 3 replicates from 2 independent experiments*.

### Preferential Bacterial Eradication

**Figure 3:**
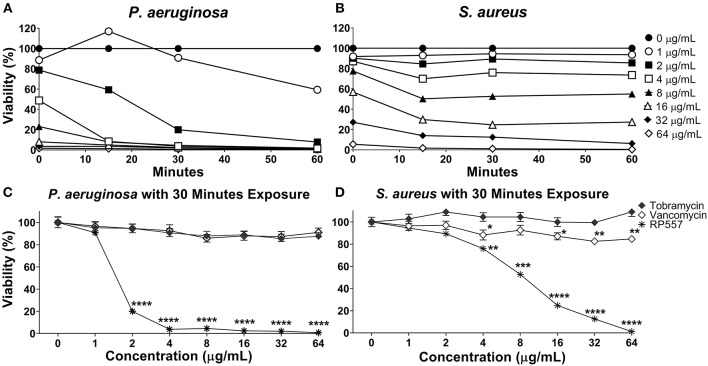
RP557 immediately destroys *P. aeruginosa* and *S. aureus* in an increasing dose-dependent manner. The bactericidal effectiveness of RP557, tobramycin and vancomycin were evaluated against bioluminescent *P. aeruginosa* 19660 **(A)** and *S. aureus* 49525 **(B)** through 60 min of exposure. The concentration dependence of RP557, tobramycin and vancomycin following 30 min of exposure with *P. aeruginosa* and *S. aureus* is shown in **(C,D)**, respectively. The bioluminescence of viable cells was quantitated non-invasively with an IVIS Lumina bioimaging system. Bioluminescence reflects bacterial viability as is directly correlated with number of colony forming units. Data represent the mean ± SE of triplicate replicates from two independent experiments; statistically significant (^*^P < 0.05; ^**^P < 0.01, ^***^P < 0.001, ^****^P < 0.0001), using one-way ANOVA followed by Tukey analysis. For some points, the error bars are shorter than the height of the symbols.

RP557 rapidly destroys *P. aeruginosa* and *S. aureus* as assessed using non-invasive continuous bioluminescence *in vitro* time-kill assays (Figures 3A,B). The marked direct bactericidal activity of RP557 relates to direct electrostatic interaction with the bacterial cell membrane. The concentration-dependent bactericidal activity of RP557 and clinically approved antibiotics, tobramycin and vancomycin, against *P. aeruginosa* and *S. aureus* is shown in Figures 3C,D, respectively following 30 min exposure. RP557 rapidly kills both Gram-negative and Gram-positive bacteria in a concentration-dependent manner, indicating that the bactericidal mode of action is via specific, and direct, membranolytic disruption. By contrast, tobramycin, an aminoglycoside which disrupts cell membranes and impairs bacterial protein synthesis in Gram-negative bacteria, and vancomycin which acts by inhibiting peptidoglycan synthesis thereby preventing cell wall synthesis in Gram-positive bacteria, exhibited no trace of antibacterial activity following incubation for 30 min.

**Figure 4:**
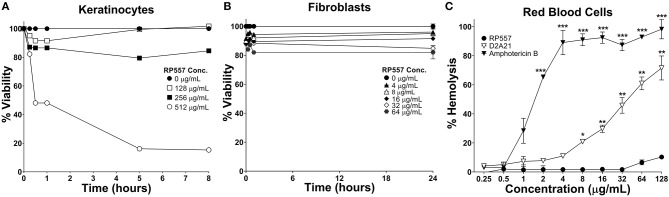
RP557 exhibits minimal cytotoxicity. Keratinocyte and fibroblast cytotoxicity was non-invasively assayed using bioluminescent strains of human HaCaT keratinocytes **(A)** and murine L929 fibroblast cells **(B)** and viability assayed using an IVIS Lumina imaging system. Hemolysis toward human RBCs is shown in **(C)**. Data represents the mean ±SE. Bioluminescent experiments were performed in triplicate from two independent experiments. The red blood cell viability assay involved two replicates; statistically significant in comparison to RP557 (^*^P < 0.05; ^**^P < 0.01, ^***^P < 0.001), using one-way ANOVA followed by Tukey analysis.

The clinical development of AMPs has been hindered by unwanted toxicity to mammalian cells (Mannis, 2002). RP557 exhibited limited toxicity against human keratinocytes, murine L929 fibroblasts and human red blood cells (Figure 4) at concentrations far greater than those required to kill bacterial cells (MICs between 4 and 8 μg/mL, Table 1). Following 8 h incubation with keratinocytes, RP557 exhibited limited cytotoxicity with no cell killing observed at 256 μg/mL (Figure 4A), and limited fibroblast cell killing observed at the maximum concentration evaluated, 64 μg/mL following 24 h incubation (Figure 4B). The concentration of RP557, D2A21, and amphotericin B to cause human red blood cell hemolysis is shown in Figure 4C. The minimum hemolytic concentration required to cause 10% hemolysis, MHC, of human red blood cells was 0.75, 4, and 128 μg/mL for the broad-spectrum antifungal amphotericin B, the earlier dAMP analog D2A21, and RP557, respectively.

### RP557 Has a High Selectivity Index

**Figure 5:**
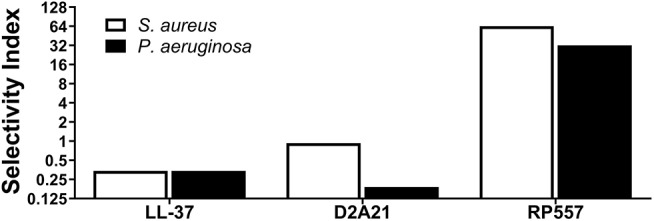
RP557 possesses potent selectivity for bacterial pathogens. The Selectivity Index is defined by the dose required to hemolyze 10% human red blood cells compared to the *S. aureus* and *P. aeruginosa* minimal inhibitory concentration (MIC).

In evaluating the clinical utility of RP557 compared to an earlier evaluated dAMP, D2A21, and the endogenous LL-37, for the prevention, and treatment, of combat wound infections, the Selectivity Index was determined. The Selectivity Index is defined as the AMP concentration which causes 10% human RBC hemolysis compared to the concentration (MIC) at which the growth of *S. aureus* and *P. aeruginosa* is completely suppressed. The larger the Selectivity Index, the greater the specificity of the AMP for destroying the bacteria. RP557 possesses a Selectivity Index ratio of 64 and 32 for *S. aureus* and *P. aeruginosa* respectively, compared to 0.93 and 0.19 for D2A21 and 0.34 for LL-37 for both pathogens (Figure 5).

### Bacteria Did Not Develop Resistance to RP557

**Figure 6:**
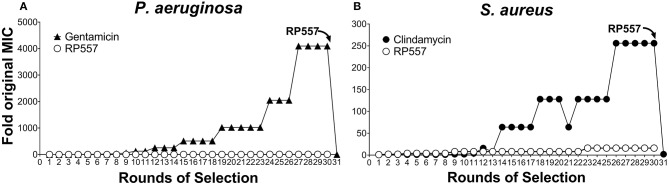
*P. aeruginosa* and *S. aureus* did not develop resistance to RP557. Sub-inhibitory concentrations of RP557, gentamicin and clindamycin were cultured with *P. aeruginosa* ATCC 27853 **(A)** and *S. aureus* ATCC 29213 **(B)** for 24 h. Bacteria showing growth in the highest concentration were re-passaged in fresh dilutions containing sub-MIC levels of each component for 30 consecutive passages; means are shown.

The rapid destruction of pathogenic cells by RP557 infer a theoretical reduced likelihood of developing bacterial resistance. However, to confirm this, RP557 and conventional antibiotics, gentamicin and clindamycin, were serially passaged against both *P. aeruginosa* (Figure 6A) and *S. aureus* (Figure 6B) at subinhibitory concentrations to ascertain whether resistance, assessed by growth at higher concentrations, occurs. Gentamicin and clindamycin developed strong resistance during serial passaging whereas RP557 did not (Figure 6). The MIC value for gentamicin during Passage #0 and Passage #1 was 0.125 μg/mL against *P. aeruginosa*, and the MIC value during Passage #30 was 512 μg/mL, representing a 4,096-fold increase in MIC value; whereas by contrast, the MIC values of RP557 remained relatively stable. The MIC value for clindamycin on Passage #0 and Passage #1 was 0.125 μg/mL against *S. aureus* while Passage #30 was 32 μg/mL, representing a 256-fold increase. In contrast, the MIC value for RP557 on Passage #0 and Passage #1 was 2 μg/mL with a corresponding MIC value of 32 μg/mL on Passage #30, representing a 16-fold increase.

Gentamicin resistant *P. aeruginosa* and clindamycin resistant *S. aureus* from passage 30, with MIC values of 512 and 32 μg/mL, were susceptible to RP557 with resultant MIC values of 4 and 2 μg/mL, respectively. Therefore, there is no cross resistance to dAMPs with the gentamicin resistant *P. aeruginosa* and clindamycin resistant *S. aureus*.

### Biofilm Disruption

**Figure 7:**
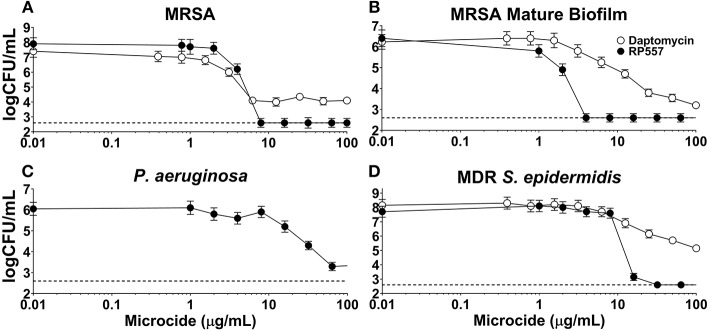
RP557 disrupts *P. aeruginosa*, multidrug resistant *S. epidermidis* and MRSA biofilms, including mature biofilm. Dose-dependent concentration response of RP557 against 6 hr MRSA ATCC 1556 **(A)**, mature (120 h) MRSA ATCC 1556 **(B)**, *P. aeruginosa* P14 **(C)**, and MDR *S. epidermidis* ATCC 700578 **(D)** biofilms. Biofilms were established in plastic 96-well plates and then treated overnight with increasing concentrations of RP557 or daptomycin and then CFUs evaluated. Three technical replicates were performed for each condition tested. The dashed lines represent the lower limit of detection (LLOD) which is 2.6 log CFU/mL.

The inhibitory effects of RP557 and daptomycin, were evaluated in both preformed biofilm and mature biofilm. RP557 completely disrupted both preformed (Figure 7A) and mature MRSA biofilm (Figure 7B) exhibiting EC_90s_ of 6 and 2 μg/mL, respectively (Figure 7). In contrast, daptomycin was only able to reduce preformed MRSA biofilm by 55% yielding an EC_90_ >> 100 μg/mL; moreover, the concentration required to reduce mature biofilm by 90% was exponentially larger at ~100 μg/mL. RP557 was able to inhibit *P. aeruginosa* biofilm, though at higher concentrations compared to MRSA, yielding an EC_50_ of 32 μg/mL (Figure 7C). RP557 completely impacted MDR *S. epidermidis* biofilm with an EC_90_ of 8 μg/mL, while daptomycin only exhibited modest activity (Figure 7D). A 100 μg/mL daptomycin concentration reduced MDR *S. epidermidis* biofilm by just 37%.

### *In vivo* Efficacy

**Figure 8:**
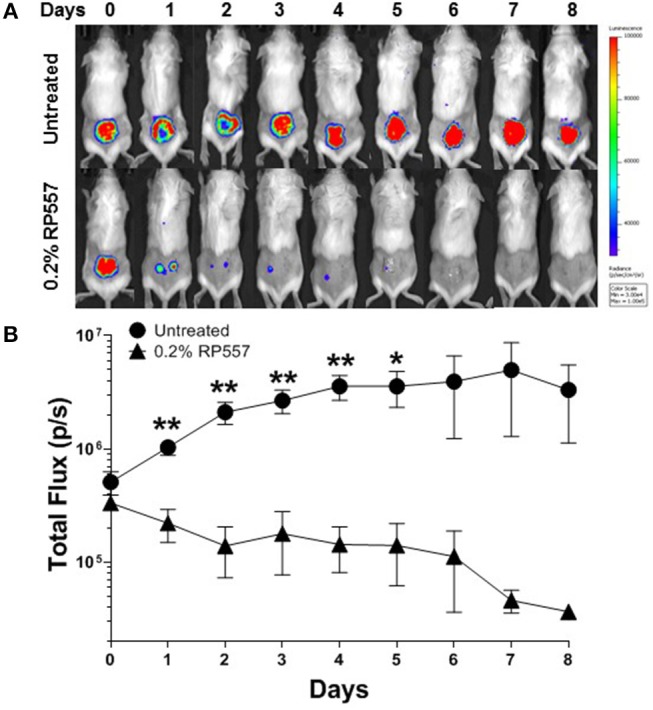
A single topical treatment of 0.2% RP557 reduced MRSA infection in a murine skin abrasion wound infection model. Infected scratch wounds were created on the backs of immunosuppressed BALB/c mice with bioluminescent MRSA (Xen31, MRSA ATCC 33591). After 4 h, 0.2% RP557 in 2% hydroxypropyl methylcellulose was applied to one group, with the other group serving as a non-treatment control. The bioluminescence of viable cells was quantitated non-invasively using bioluminescence imaging **(A)** and RP557 bactericidal effectiveness measured by the magnitude of the bioluminescent signal **(B)**. Data is expressed as mean ± SE of 5 mice, except for the untreated control group where an animal was found dead on Day 4. Statistical significance, compared to untreated control, determined by two-tailed unpaired-*t* test (^*^p < 0.05, ^**^p < 0.01).

To emulate a cutaneous MRSA wound infection, cuts were introduced into the backs of immunocompromised BALB/c mice and the wounded area was then inoculated with bioluminescent MRSA bacteria. Bioluminescent MRSA (Xen31, derived from the parental strain *S. aureus* ATCC 33591) has a stably integrated lux operon that leads to spontaneous light emission at 37°C in the absence of any exogenously added substrate. MRSA CFUs correlate linearly with the bioluminescence emitted by the MRSA bacteria (Francis et al., 2000; Grinholc et al., 2015). RP557 demonstrated statistically significant *in vivo* activity in eradicating MRSA in this abraded infection murine model (Figures 8A,B). A single topical application of 0.2% RP557 significantly reduced MRSA from untreated control levels (*p* < 0.01 through Day 4 and *p* < 0.05 on Day 5) with the bioluminescence returning to baseline levels around Day 7. Topical 0.2% RP557 application also had a surprisingly favorable systemic effect on the infected animals. Animals had received cyclophosphamide to become immunocompromised and as a result had lost body weight. On Day 0, prior to RP557 treatment, there was no difference in body weight between the two immunocompromised groups however, following dAMP treatment, on Days 1 through 4 there was a statistically significant difference in body weight between the two groups. Topical RP557 application decreased body weight loss; for example, on Day 3 the control animals had lost 17.33 ± 2.06% body weight compared to 7.20 ± 1.37% for the RP557 treated group, *P* < 0.01.

## Discussion

RP557 is a water-soluble, chemically stable amphipathic dAMP that exhibits broad-spectrum activity against both Gram-negative and Gram-positive clinical isolates and fungi. Bacterial eradication should be the primary goal of antibiotic treatment, with bacterial load the main determinant of therapeutic outcome (Ball et al., 2002). Rapid, precise healing at the epithelium is critical for restoration of the skin's important protective function, and should limit the emergence of resistance and the spread of infection. Antimicrobial agents that disrupt cell membranes or interfere with essential enzyme function are likely to be bactericidal, whereas agents that inhibit ribosomal function are most likely bacteriostatic (Finberg et al., 2004). *In vitro* time-kill assays, utilizing bioluminescent *P. aeruginosa* and *S. aureus*, underscored the rapid effectiveness of RP557 at relatively low doses (2 μg/mL).

An efficacious antimicrobial must be able to selectively inhibit and kill bacteria. The development of a clinically viable dAMP has been hampered by unwanted toxicity to mammalian host cells at therapeutic doses (Mannis, 2002; Edwards et al., 2017). Therefore, the cytotoxicity of RP557 was evaluated in murine fibroblast cells and with human RBCs and keratinocytes. At doses of RP557 that kill bacteria upon contact, no mammalian cytotoxicty was observed. This is in stark contrast to the progenitor peptides that induced toxicity to mammalian host cells; the minimum hemolytic concentration that caused 10% hemolysis for Tachyplesin 1 and D2A21 was 0.25 μg/mL (Edwards et al., 2017) and 4 μg/mL, respectively, compared to 128 μg/mL for RP557. As a corollary, limited cytotoxicity was observed following 24 h RP557 incubation with murine fibroblasts at the highest concentration evaluated (64 μg/mL); and following 8 h incubation at 256 μg/mL RP557 with human keratinocytes, greater than 80% viability was observed. The calculated Selectivity Index for RP557, defined as the ratio of doses at which RP557 is active against bacteria without inducing cytotoxic damage to the surrounding mammalian cells, demonstrated a relative safety margin greater than 128.

*P. aeruginosa* and *S. aureus* did not become resistant to sub-inhibitory concentrations of RP557 after 30 rounds of selection whereas gentamicin and clindamycin did, as evidenced by growing in antibiotic concentrations at 4,096 and 256 times the MIC, respectively, after 30 days. Gentamicin resistant *P. aeruginosa* and clindamycin resistant *S. aureus* from passage 30 with MIC values of 512 and 32 μg/mL were susceptible to RP557 with resultant MIC values of 4 and 2 μg/mL, respectively. Therefore, there is no cross resistance to dAMPs with the gentamicin resistant *P. aeruginosa* and clindamycin resistant *S. aureus*.

Biofilms are associated with the persistence of military wound infections (Akers et al., 2014). Conventional antibiotics are most effective against actively metabolizing cells and fare poorly against persister cells. RP557's direct perturbation of cellular membranes is expected to be more effective at eradicating biofilms (Woodburn et al., under review). Daptomycin, a lipopeptide last-resort drug used for treatment of *S. aureus* infection shows activity against biofilms formed by MRSA (Siala et al., 2014). RP557 completely disrupted both preformed and mature MRSA biofilm exhibiting EC_90_ of 6 and 2 μg/mL, compared to over >>>100 μg/mL for daptomycin.

*S. epidermidis* is a major nosocomial pathogen and is amongst the most prevalent bacteria of the human skin and mucous membrane microflora, and therefore presents a major problem in the treatment of infections involving biofilm formation, including those related to devices. RP557 was able to completely eradicate MDR and methicillin-resistant *S. epidermidis* biofilm with an EC_90_ of 8 μg/mL while daptomycin exhibited moderate activity. A 100 μg/mL daptomycin concentration reduced MDR *S. epidermidis* biofilm by 37% and methicillin-resistant *S. epidermidis* biofilm by 41%. Furthermore, biofilm formation is a key driver of fungal pathogenicity (Sherry et al., 2017). RP557 was remarkably effective in killing fungi in preformed *C. albicans* biofilm and preventing biofilm formation, while fluconazole was markedly ineffective (Woodburn et al., under review). Furthermore, scanning electron microscopy on planktonic and on preformed biofilm *C. albicans* suggested that dAMP mediated fungal damage was via membrane perturbation (Woodburn et al., under review). RP557 completely eliminated *MRSA* infection following a single topical application of 0.2% using a clinically relevant murine MRSA wound infection model. Infection was monitored over an 8-day period with bioluminescent imaging.

Despite much promise and the dire need for new anti-infectives, AMPs have previously not garnered appeal due to apparent high cost, proteolytic susceptibility and concerns relating to cytotoxicity (Mulani et al., 2019). However, recent advances in solid phase peptide chemistry have greatly reduced manufacturing costs; and as shown here, the recently engineered RP557 exhibits robust stability, broad spectrum effectiveness against Gram-negative and Gram-positive bacteria and fungi, substantial specificity for bacteria over mammalian cells, reduced likelihood of developing pathogen resistance, prevention of biofilm formation, effectiveness against established biofilm, and activity against MDROs. The clinical implementation of RP557 could prove a novel method of dealing with antibiotic resistance while at the same time providing a new broad-spectrum treatment against wound pathogens and biofilm infections in both military and civilian contexts.

## Data Availability

All datasets generated for this study are included in the manuscript and/or the supplementary files.

## Ethics Statement

This study was carried out in accordance with the recommendations the Guide for the Care and Use of Laboratory Animals (NIH Publication, 2011). The protocol was approved by the Institutional Animal Care and Use Committee at Lumigenics Inc.

## Author Contributions

KW, JJ, and LC contributed conception and design of the study. KW and LC organized the database, performed the statistical analysis, and wrote the first draft of the manuscript. KW, JJ, and LC wrote sections of the manuscript. All authors contributed to manuscript revision, read and approved the submitted version.

### Conflict of Interest Statement

KW, LC, and JJ own stock in Riptide Bioscience, Inc.
